# Cat Gets Its Tern: A Case Study of Predation on a Threatened Coastal Seabird

**DOI:** 10.3390/ani9070445

**Published:** 2019-07-16

**Authors:** Claire N. Greenwell, Michael C. Calver, Neil R. Loneragan

**Affiliations:** 1 Environmental and Conservation Sciences, Murdoch University, 90 South Street, Murdoch, Western Australia 6150, Australia; 2 Centre for Sustainable Aquatic Ecosystems, Harry Butler Institute, Murdoch University, 90 South Street, Murdoch, Western Australia 6150, Australia

**Keywords:** *Felis catus*, Laridae, local extinction, predator control, species decline, Sternidae, *Sternula nereis*, wildlife conservation

## Abstract

**Simple Summary:**

Feral cats living completely independently of people are, unequivocally, agents of wildlife decline, which is linked to the local extinction of numerous bird, mammal and reptile species worldwide. However, evidence of the impact of pet and semi-feral (stray) cats on wildlife is somewhat limited, possibly due to the rapidity with which predation events occur. This study highlights the impact of a semi-feral cat on a threatened seabird colony of Australian Fairy Terns, *Sternula nereis nereis*, in Mandurah, south-western Australia. Evidence for significant predator-induced mortality, the alteration of the natural behavior of nesting birds in response to a persistent predator, and the complete reproductive failure of 111 nests—due, largely, to predation by a single, desexed, semi-feral cat—is presented. Trap-neuter-release was proposed as a humane response to tackle cat overpopulation but it fails to address the recurrent depredations on native wildlife that occur post-release. This case-study demonstrates that desexed, free-roaming cats remain a significant threat to wildlife and can lead to swift population declines and the local extirpation of native species.

**Abstract:**

Domestic cats have a cosmopolitan distribution, commonly residing in urban, suburban and peri-urban environments that are also critical for biodiversity conservation. This study describes the impact of a desexed, free-roaming cat on the behavior of a threatened coastal seabird, the Australian Fairy Tern, *Sternula nereis nereis*, in Mandurah, south-western Australia. Wildlife cameras and direct observations of cat incursions into the tern colony at night, decapitated carcasses of adult terns, dead, injured or missing tern chicks, and cat tracks and scats around the colony provided strong evidence of cat predation, which led to an initial change in nesting behavior and, ultimately, colony abandonment and the reproductive failure of 111 nests. The death of six breeding terns from the population was a considerable loss for this threatened species and had the potential to limit population growth. This study highlights the significant negative impacts of free-roaming cats on wildlife and the need for monitoring and controlling cats at sites managed for species conservation. It also provides strong evidence against the practice of trap-neuter-release programs and demonstrates that desexed cats can continue to negatively impact wildlife post-release directly through predation, but also indirectly through fundamental changes in prey behavior and a reduction in parental care.

## 1. Introduction

Domestic cats have been globally distributed by people as companion animals and vermin controllers, and different categories of cat populations are described by their interactions with people. These descriptions vary with locale, but those of Baker et al. [1] and Cove et al. [2] are useful for assessing potential interactions with wildlife. Based on these descriptions, feral cats exist without any human support, semi-feral cats are at least partially provisioned by people (deliberately or incidentally through garbage), pet cats (indoor/outdoor housecats in Cove et al. [2]) are owned and supported by their household but roam mainly without restriction, while housebound cats (only in Baker [1]) remain on their owners’ properties at all times. These distinctions are important ecologically because the survival, reproduction, diet, and ranging behavior of pet cats and semi-feral cats are mediated by humans [1]. Furthermore, differences in behaviors, such as the depredation of wildlife, might be marked between the different groups of cats, demonstrating the need for management options on an individual basis [2,3].

Predation by feral cats is a major agent of extinction, second only to rodent predation [4]. Feral cats are linked to the extinction of at least 63 bird, mammal and reptile species, and threaten another 420 species worldwide [4], contributing to declines or local extinctions of native fauna in settings ranging from islands [5] to restricted mainland environments [6,7,8], including their implication in the extinction of numerous Australian mammals and the threatened status of other extant species [9]. Seabird populations are amongst those threatened by cats, invariably at terrestrial nesting sites because seabirds often lack the behavioral, life-history and morphological defenses required to overcome mammalian predators [10,11,12].

However, the contribution of pet cats and semi-feral cats to fauna decline, including that of nesting seabirds, remains controversial [13,14]. For example, in the United Kingdom, the Royal Society for the Protection of Birds claims that ‘despite the large numbers of birds killed by cats in gardens, there is no clear scientific evidence that such mortality is causing bird populations to decline’ [15]. In contrast, across the Atlantic, the American Bird Conservatory concludes that ‘although domestic cats (*Felis catus*) can make wonderful pets, they threaten birds and other wildlife and disrupt ecosystems’ [16].

The urban, suburban and peri-urban environments where pet cats and semi-feral cats are concentrated are important for biodiversity conservation, providing shelter and food for resident and migratory wildlife, including seabirds, in the face of habitat destruction for agriculture, housing or other human uses elsewhere [17,18,19]. Cats are popular pets in these environments, and frequently, populations of semi-feral cats are found roaming freely, often supported by provisioning from people [20,21]. Both pet and semi-feral cats may hunt wildlife, spread disease, or create sub-lethal effects through fear (see short review in Calver et al. [14]). While mortality caused by pet and semi-feral cats is undeniable, cases where documented reductions in wildlife populations occur as a result of cat predation are sparse, although examples include some lizards [22,23], mammals [24], and birds [25]. Previous studies correlating the densities of pet cats with prey densities or simple presence/absence were inconclusive, with some claiming effects [26] and others not [13].

**Figure 1:**
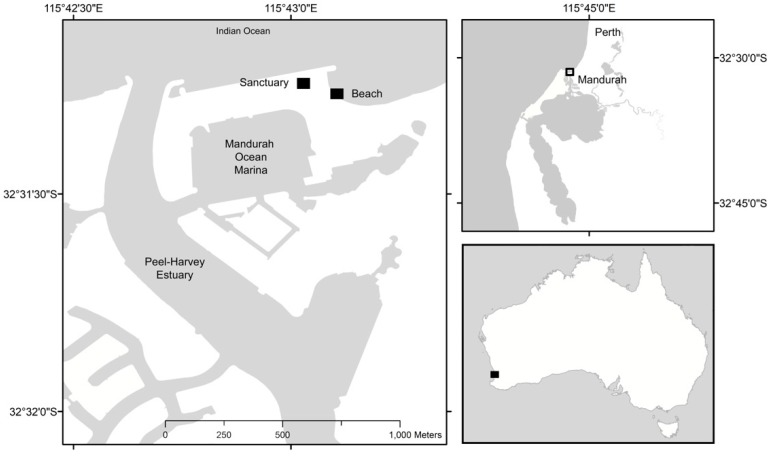
Map showing the location of the Fairy Tern, *Sternula nereis nereis*, sanctuary and beach colonies established in Mandurah, Western Australia during the 2018 breeding season (October to December).

One possible explanation for such divergent assessments of the evidence is that the effect of cat predation is swift, so there may not be an adequate opportunity to detect declines or local extinctions [23]. Here, we report evidence for such a rapid effect caused by a semi-feral cat preying on a nesting colony of ~220 Fairy Terns, *Sternula nereis nereis*, a threatened Australian seabird, in a suburban setting in Mandurah, Western Australia (Figure 1). Although the observations of cat predation were opportunistic, because our study was focused on the breeding biology of the terns, the effects were compelling.

## 2. Materials and Methods 

### 2.1. Study Species 

The Australian Fairy Tern is endemic to Australia and is one of two small terns (*Sternula*) to breed in this country (the other being the little tern, *Sternula albifrons*). Like most seabirds, Fairy Terns nest in colonies and their breeding sites are usually located on shorelines, coastal lagoons, salt lakes, and in the lower reaches of estuaries where their main prey, small schooling fish (baitfish) such as sardines, sandy sprat (both Clupeidae) and silversides or hardyheads (Atherinidae), are naturally abundant [27]. Human impacts, including coastal development and intense use of near-shore environments have made many former colony sites unsuitable for the formation of breeding aggregations. These impacts, coupled with predation by domestic and feral animals, have reduced recruitment and adult survivorship over the past few decades [27,28]. Following an estimated 24% population decline in eastern Australia between 1974 and 2007 and no evidence that their main threats to survival were abating, Fairy Terns were listed as vulnerable in 2011 by the Australian government [28,29]. In the Conservation Advice, developed in accordance with s266B of the Environment Protection and Biodiversity Conservation Act 1999 (Australian Commonwealth), predation by cats and foxes was noted as a major threatening process for nesting Fairy Terns [30].

### 2.2. Study Site and Colonies

Sites managed specifically for a target species have frequently been utilized for tern conservation around the world [31,32] and are areas where planned management interventions, such as predator control and habitat enhancement, are used to maintain local aggregations of breeding birds and enhance nesting success. The managed sites are typically located in areas where coastal development and human population pressures have greatly reduced the number of secure, natural nesting areas. In Western Australia, managed sites are being used to overcome a lack of suitable nesting habitats available to Fairy Terns in historically important breeding areas.

The mouth of the Peel-Harvey estuary in Mandurah is an important breeding location for Fairy Terns due to its close proximity to a reliable supply of baitfish (Figure 1) [33]. However, the development of the Mandurah Ocean Marina (Stage 1 completed in 2001) has eliminated former nesting locations and the low elevation of other potential sites on the estuary has, in recent times, resulted in persistent breeding failure due to colony inundation during storm surge events [33]. In 2017, a parcel of land on Breakwater Parade in the Mandurah Ocean Marina, ‘the sanctuary’, was set aside to facilitate breeding attempts by Fairy Terns in the region (Figure 1). The site was cleared, a 1.2-m-high chain mesh fence lined with shade-cloth was installed to limit human and vehicular traffic, and a layer of shell material was spread over the ground surface to replicate the preferred nesting habitat of Fairy Terns.

Town Beach is a second nesting site nearby. It is located along Breakwater Parade, Mandurah, ~50 m from the sanctuary and is of high recreational importance for swimming, walking and relaxing. The beach is artificially widened between June and November through a sand by-passing scheme used to ensure safe navigation into the Mandurah channel [34]. Between June and November each year, the sand pumping scheme moves 160,000 m^3^ of sand from the south of the entrance channel to Town Beach (600 m north), where it is deposited in the form of a slurry [34]. During the 2018–2019 breeding season, Fairy Terns nested on an artificially-widened section of Town Beach, near the sand by-passing outlet (Figure 1).

### 2.3. Monitoring of the Colonies

Observations of the Fairy Terns in and around the sanctuary began in anticipation of nesting on 3 October 2018 and continued until 1 January 2019 on most days, usually in the three hours following sunrise and/or prior to sunset. A spotting scope (Swarovski ATS 65, 20–60×) and binoculars (Swarovski 10 × 32) were used to observe nesting terns from a vantage point outside the fenced area to minimize disturbance to the birds. Observations of disturbance were noted on a daily basis, and nest and chick counts were recorded on an ad-hoc basis. Nest counts were quantified by counting the number of sitting birds (i.e., birds sitting in an incubating posture, indicating the presence of an egg), as opposed to nests with eggs, to overcome the potential for disturbance. Overnight observations were made between 19:00 and 05:00 on 1 to 5 December, and 11 to 16 December, inclusive, to observe whether nocturnal predators, i.e., cats, were disturbing the colony at night and to prevent incursions by any cats. Sunrise-to-sunset observations of nesting birds were made between 21 and 28 November. The observations were continuous, except for a 30-min period between 12:30 and 13:30.

On 24 October, two Spartan 3G wildlife cameras were installed in the sanctuary by the City of Mandurah to monitor the nesting birds. The wildlife cameras were serviced and repositioned on 27 November. Following observations of cat incursions, ten ultrasonic cat deterrents (On-Guard Mega-Sonic Cat Repeller^©^ (Defenders, Norfolk, United Kingdom, www.stvpestcontrol.com) were installed in a circle around the periphery of the colony on 27 November, in an attempt to prevent incursions by cats into the sanctuary. The devices include infra-red motion detectors (with a coverage of up to 12 m in a 98° arc), which, when triggered, emit an ultrasonic sound disliked by cats, effectively reducing the frequency and duration of cat visitations [35].

The beach-nesting colony was roped off by the City of Mandurah on 1 November to deter incursions by beachgoers in an attempt to reduce disturbance of the terns. The area was completely fenced off using a shade-cloth barrier on 13 December 2018 to prevent flightless chicks from wandering down the beach. The observations of this colony were made as outlined above for the terns in the sanctuary.

Observations on an ad-hoc basis and those conducted between 21 and 28 November were conducted in accordance with Murdoch University Animal Ethics Committee (AEC) Approval (Protocol 546, Permit RW3077/18). All other activities and management actions were conducted by the City of Mandurah, the managers of the land, whose actions were not subject to AEC approval. Volunteers of the Western Australian Fairy Tern Network aided in the monitoring of the colonies under approval from the City of Mandurah.

## 3. Results

### 3.1. Sanctuary Colony

**Figure 2:**
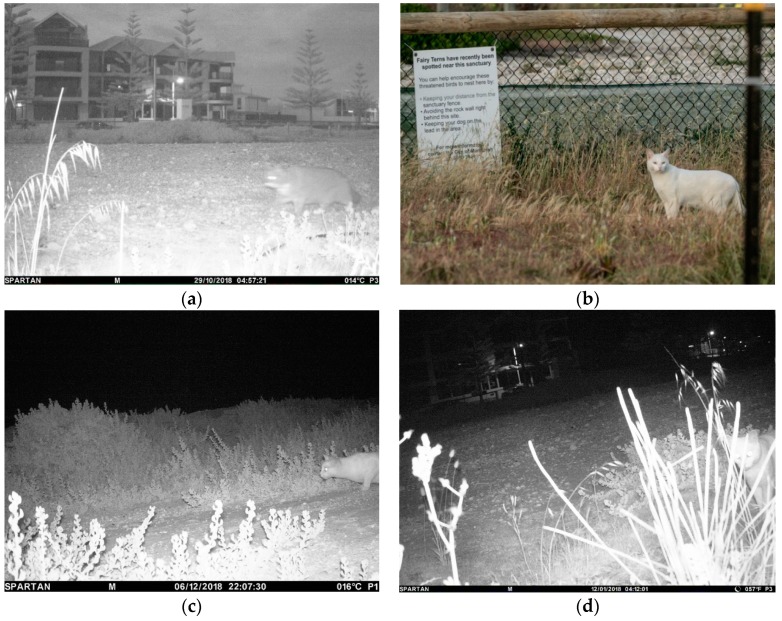
Photographs of a pet and semi-feral cat in and around the Fairy Tern Breakwater Parade sanctuary, taken by wildlife cameras ((**a**,**c**,**d**) City of Mandurah, 2018) and a local resident ((**b**) England, L., 2018).

On 29 October, the Fairy Terns had established ~12 nests and began incubating their eggs continuously in the sanctuary (for details see [32]), peaking at 111 nests by mid-November (Table 1). On 29 and 30 October, a grey pet cat was photographed by wildlife cameras on two separate occasions each night, but the cat was subsequently trapped by the City of Mandurah rangers on 23 November and impounded (Figure 2a). The cat was registered and microchipped, and the rangers requested the cat be kept indoors for the remainder of the nesting season. No further incursions by this cat were recorded and there was no evidence of predation by this cat (Table 2).

Between 18 November and 11 December, local residents and wildlife cameras recorded a white cat, with bi-colored eyes, in and around the sanctuary (Figure 2b). The cat is believed to have entered the colony on at least six separate nights, sometimes on several occasions during any one evening (Figure 2c,d). A photograph taken by a resident from a second-floor balcony on 30 November shows a white cat in the center of the nesting area, crouched and possibly eating. After incursions into the colony by the cat, cat foot-tracks and scats were observed in and around the sanctuary, carcasses of six decapitated Fairy Tern adults were found, and at least 40 chicks were found missing, injured or dead (Table 2).

The City of Mandurah sent out two letters to the surrounding residents during the Fairy Tern nesting season, requesting that pet cats be kept indoors and advised that cat trapping was being carried out. After the letters were sent out, a single pet (registered and microchipped) cat was trapped ~1 km from the sanctuary (Table 2) but there was no other evidence of pet cats roaming in or near the sanctuary.

On 8 December, the terns were highly unsettled and appeared to have lost social cohesion. Parental care was somewhat reduced, with adult terns spending less time on the ground guarding their chicks. The social group defense mechanisms of the terns appeared to have broken down, facilitating predation of chicks by a single Nankeen Kestrel, *Falco cenchroides* (Table 1). By 11 December, the colony had been abandoned, except for seven chicks that were being fed by adults but were eventually taken by the kestrel over several days (Table 2).

On 12 December, the white cat was captured. The cat was not microchipped or collared, but it was desexed. Reports from local residents suggest that the cat was, possibly, living in an abandoned house and bushland in the area (Table 2).

### 3.2. Beach-Nesting Colony

The first eggs from three nests were laid on 30 October and the colony peaked at ~40 nests by mid-late November (Table 1). The beach-nesting colony faced a range of threats, including incursions into the roped area by people and dogs (despite it being a dog-free beach) and predation of eggs by Silver Gulls, *Chroicocephalus novaehollandiae*, particularly during the early egg-laying period. Perhaps the most significant threat faced by the terns was erosion of the beach due to the cessation of the annual sand by-passing scheme in early November. Beach erosion forced several Fairy Terns to abandon their nests. However, in some cases, the nests were saved by incrementally moving the eggs a short distance (~15 cm) up the beach, where they were subsequently incubated (Corker, C. 2018, Western Australian Fairy Tern Conservation Network, pers. comm.) (Table 3).

The first chick hatched on 5 December and by 8 December, at least 8 chicks had hatched. The terns continued their incubation and parental care routines after the sanctuary colony was abandoned (11 December). After the sanctuary colony lost social cohesion, the kestrel established a pattern of feeding on chicks and became an efficient chick-predator. Once no more chicks remained in the sanctuary, the kestrel depredated chicks from the beach, despite desperate attempts by the Fairy Terns to warn off the raptor. It is possible that the chicks were silhouetted against the shade-cloth fencing, enabling the kestrel to locate its prey more efficiently (Corker, C. 2018, Western Australian Fairy Tern Conservation Network, pers. comm.) (Table 3).

As the chicks became mobile, the adults moved the chicks up onto the nearby sea wall, some 40 m away from the nesting area. By 1 January 2019, no Fairy Terns remained on the beach. Two juveniles from the sea wall are believed to have fledged successfully (Table 3).

## 4. Discussion

The observations of cat depredation on Fairy Terns were opportunistic, given that the aim of the study was to encourage breeding by Fairy Terns and not on the potential impacts of domestic cats *Felis catus*. Nevertheless, wildlife cameras and direct observations of cat incursions into the tern colony at night, decapitated carcasses of adult terns, dead, injured or missing tern chicks, and cat tracks and scats around the colony provided strong evidence of cat predation. Changes in nesting behavior followed quickly, but ultimately the colony was abandoned and 111 nests failed. The death of six breeding terns from the population is a considerable loss for this threatened species and has the potential to limit population growth. A nearby colony that suffered multiple disturbances, but no cat depredations, still managed to fledge a small number of chicks. Thus, free-roaming cats can cause significant impacts on wildlife populations, including local extirpation. These can be so rapid that they are easily overlooked.

### 4.1. Invasive Predators and Seabird Decline

Global seabird populations have been in decline in recent decades, with 28% (n = 97 species) of all seabird species now classified as threatened with extinction and a further 10% (n = 35 species) near threatened by the International Union for Conservation of Nature [10,37]. Invasive species, such as Black Rats, *Rattus rattus*, and cats are the most pervasive threat to seabirds, negatively impacting an estimated 75% (n = 73) of all threatened seabird species, almost double that of any other known threat (e.g., problematic native species, human disturbance and fisheries by-catch [10,38]). Where the survival of breeding adults is threatened directly, such as by the presence of a cat, nests are temporarily abandoned until the threat subsides, and in extreme cases, eggs and chicks may be abandoned altogether [11,39]. The extirpation of large seabird populations by cats has been well-documented on numerous oceanic islands, with relict populations persisting only in locations free from feline predators [40,41,42]. The eradication of cats from islands has demonstrably improved conservation outcomes for seabird populations through a reduction in adult and juvenile mortality, allowing the recolonization of former colony sites [40,42,43,44]. However, in some cases, the explosion of rat populations following cat removal has hampered recovery due to persistent egg and chick predation by rats [38,44,45].

These generalizations are illustrated by two specific examples. A study of cat predation on Black-vented Shearwater, *Puffinus opisthomelas*, on Natividad Island, Mexico found that a 2.5 kg cat was estimated to consume ~328 g of food per day, of which shearwaters made up 295 g (90%) of the total diet [12]. Up to 1.5 shearwaters were estimated to be consumed per cat per day, or 40.5 shearwaters per month. In a one-month period, the population of 25 cats on the island was largely responsible for the deaths of 1012 shearwaters [12]. This equates to an estimated 5% decline in the annual growth rate of shearwaters on Natividad Island for every 20 cats in a population of 150,000 birds [12]. Cats were also implicated in the swift demise of the endangered Black-fronted Tern, *Sterna albostriata*, on the Rangitata River, New Zealand. Tracks of a single cat, found imprinted into the sand, showed that the cat had moved systematically from nest to nest, leaving behind a trail of destruction [11]. Fresh scats, characteristic of a cat, were also found next to two of the nests [11]. Twelve of the 28 active nests showed direct evidence of predation (e.g., broken eggs), and the carcasses of six adult terns were found, three of which had been decapitated [11]. The failure of the terns to respond to the presence of a nocturnal predator, such as a cat, may be related to the stealth with which cats hunt, and the limited vision of some birds in extreme darkness [46,47,48].

### 4.2. The Mandurah Tern Colonies

The reproductive failure and mortality of adult Fairy Terns at Mandurah followed the pattern of the specific examples above and also illustrates the general principles of the impacts of cats on seabirds. As beach-nesting birds, Fairy Terns face significant challenges throughout the breeding season. Egg-burial and colony inundation from extreme spring tides and summer storm events are common. However, second attempts at reproduction and the re-laying of eggs commonly occurs if the first clutch is lost early in the season (at alternative locations, subject to local prey availability) [39,49,50]. This feature of their life-history, combined with their potentially long lifespan (at least 22 years [Dunlop, J.N.]; Australian Bird and Bat Banding Scheme, pers. comm. 2018) provides a buffer against individual nest failure, with the potential for replacement achieved over numerous nesting attempts within the birds’ lifetime [32,51,52,53]. The death of six breeding adults from the Fairy Tern population was, however, a considerable loss for this long-lived k-strategist. Adult survival is the most important factor influencing population growth rates and growth may be further complicated by the fact that not all pairs contributed equally to the next generation [54,55]. The successful brooding of eggs through to fledging is influenced by a range of factors, including mate quality, age, and the experience of the pair, and reproductive success is typically higher among older, more experienced pairs than younger birds [54,56,57,58]. Therefore, the loss of experienced, high-quality birds from the population is likely to have a negative effect on population growth in the longer-term.

The example reported in the present study supports the experience of Bamford and Calver [23] with the lizard *Ctenotus fallens* and the hypotheses of Grayson et al. [59] and Lilith et al. [60] for bird and mammal communities, respectively, that the effect of cat predation can be rapid and that pet cats or semi-feral cats can be responsible for significant mortality in a short time. This case study also provides support for Bonnington et al. [61], who hypothesized that the presence of a cat may detrimentally change prey behavior and, subsequently, induce indirect lethal effects by other species due to a reduction in parental care [61]. In the current study, predation (either direct or indirect) by a single semi-feral cat, resulted in the deaths of at least 40 chicks and six breeding adults from a colony of 111 pairs over a period of only 23 days, and the effective predator defenses of the colony against a small raptor (Nankeen Kestrel, *Falco cenchroides*) broke down. Aerial group defense tactics, i.e., social mobbing, are an important anti-predator strategy undertaken by many terns and gulls in an attempt to cause confusion and drive off potential predators [39,62]. However, the social cohesion necessary for this behavior was lost by early December, following the loss of adults and chicks to cat predation. While cases of dead, missing and injured chicks were observed, it is unclear whether all chick deaths were the result of direct predation in excess of what could be eaten immediately (surplus killing) or indirect effects arising from temporary nest abandonment while the cat was in the colony. When adult terns are threatened directly, nests are routinely left unattended until the threat subsides [62,63]. Therefore, it is possible that very small chicks died from exposure, i.e., nests were left so long that the chick’s body temperature cooled to below a level that was able to be thermoregulated [63].

Significantly, the main cat involved in the current study did not appear to be owned but was desexed, so it was probably a pet at some stage of its life. While cat owners in the vicinity were very responsible and restricted the roaming of their pets during the breeding period of the colony as requested, one semi-feral cat continued to hunt, and surplus killing, in which more prey were killed than could be consumed (also noted for cats in relation to depredation on tern species outside Australia, e.g., Sooty Terns, *Sterna fuscata* [64] and domestic chickens in Cuba [65]), as well as changes in the anti-predator behavior of parent birds, caused the collapse of the colony. Attempts to protect the colony using sonic deterrents proved unsuccessful, possibly due to the strong attraction stimulus provided by the colony, despite the effectiveness of such devices in other suburban settings [66,67]. Alternatively, the white cat with a single blue eye in this study may have been congenitally deaf [63,68,69], rendering the sonic devices ineffective, as was found by Crawford et al. [67]. This interaction between a single cat and a Fairy Tern colony provides important lessons for managing seabird colonies and also for managing pet cats and semi-feral cats to protect wildlife.

### 4.3. Management Implications

To overcome the effects of invasive predator-induced mortality, predator monitoring (e.g., the use of wildlife cameras, traps and direct observations) and control at sites managed for species of conservation significance is critical. As demonstrated in the present study and other studies from around the world, failure to adequately control predators can be disastrous [11,12,42,70]. The evidence for the serious negative impacts of cats on seabird populations is substantial and described as a major contributor to nest failure e.g., [3,7,9,12,54,55]. The persistence of the beach-nesting colony in the absence of cat predation highlights the significance of perceived adult survival-risk and the extent of disturbance that a colony will endure before abandoning a breeding attempt altogether.

Regarding the management of pet cats, the quick, positive response of owners to the issue of urgent wildlife protection was encouraging. Surveys have shown that approximately 60% of Australian cat owners accept that pet cats killing wildlife in cities, towns and rural areas is a problem [71], which gives some basis to encourage wildlife-friendly cat husbandry. However, it may be even more effective to highlight to owners the cat welfare benefits that arise when cats are kept on their owner’s property at all times e.g., [56]. With regard to semi-feral cats, some Australians and citizens of other countries, such as the USA, support or practice trap-neuter-release (TNR), in which semi-feral cats are trapped, desexed, and then returned to the environment as a humane response to cat overpopulation [21,72,73]. There is, nevertheless, concern and evidence that TNR cats are detrimental to wildlife populations in the USA [74]. The severe depredations caused by a single semi-feral cat in the current study are a further clear example of the consequences for wildlife management that can result if such practices are adopted widely because desexed cats still hunt post-release [67,75], as was the case for the desexed, free-roaming cat in this study. Removal campaigns, including targeted adoption where possible, would be more effective in reducing attacks on wildlife [35], especially if accompanied by restrictions on the roaming of owned cats. Our study also indicates that some habitats and some species may be especially vulnerable to cat predation and that impacts, including local extirpation, may be swift.

## 5. Conclusions

This experience generates important messages for the management of cats to protect wildlife. Regulations to restrict the movements of pet cats to their owners’ property and increased control of semi-feral and feral cats will, undoubtedly, improve conservation benefits for a diverse range of species utilizing urban, suburban and peri-urban environments. Trap-neuter-release programs should be strongly contested due to the high ongoing potential for desexed cats to negatively impact wildlife post-release, both directly and indirectly, as highlighted in the current study. Finally, targeted education programs that highlight the welfare benefits that arise from restricting pet cat movement and encourage wildlife-friendly cat husbandry, implemented at community- and national-levels, could be used to drive change in attitude and behavior among cat owners.

## Figures and Tables

**Table 1:** Summary of threats, colony development, mortality and fledgling success of nesting Fairy Terns at the sanctuary and beach-nesting colonies in Mandurah during 2018.

Indicator	Sanctuary Colony	Beach-Nesting Colony
Threats	Predation of adults and chicks by cat, predation of chicks by Nankeen kestrel ^1^.	Predation of chicks by Nankeen Kestrel ^1^, predation of eggs by Silver Gull ^2^, kestrel and dogs seen in and near colony, incursions by humans, beach erosion
Max no. of nests	111	44
Adult Fairy Tern mortality	6	0
Chick mortality	40+	0
Fledglings	0	2

^1^*Falco cenchroides*; ^2^*Chroicocephalus novaehollandiae*.

**Table 2:** Timeline showing colony development at the sanctuary in Mandurah, incursions by a pet and semi-feral cat, depredation events and management activities undertaken during the 2018 breeding season. The grey shading shows the period in which sunrise-to-sunset observations of the nesting colony were made.

Date	Timeline of Events
29 Oct 2018	First night that birds were observed incubating eggs at night see [32]. Wildlife cameras took photos of a grey pet cat in the sanctuary at 0457 and 0503 hrs. There was no evidence of predation (Figure 1a).
30 Oct 2018	Wildlife cameras took photos of grey pet cat at 00:22, 00:32, 03:52 (Figure 1a).
9 Nov 2018	~58 nests in the sanctuary
18 Nov 2018	Residents in the apartments opposite the sanctuary heard a disturbance in the colony-the terns were calling loudly. A white cat was chased out of the sanctuary between 19:00 and 20:00. There was no evidence of predation and the camera batteries had, unknowingly, gone flat.
19 Nov 2018	The rangers commenced cat trapping. Trap was set~18:00.
21 Nov 2018	The first chick observed, estimated at~2 days old.
22 Nov 2018	~95 nests in the sanctuary
23 Nov 2018	A registered, grey pet cat was found in the trap in the morning and impounded by rangers. The cat belonged to a resident living in an apartment across the road. The cat’s owner was asked to keep their cat indoors. A letter was sent out to residents requesting that cats be kept indoors until the end of the Fairy Tern breeding season. 2 nests with chicks (one with 2 chicks). At sunset, chicks from both nests alive and appear to be strong.
24 Nov 2018	2 chicks from the second nest observed dead at first light. Possible, unconfirmed colony disturbance at night forcing adults to leave chicks unattended. ~110 nests in colony. 7 nests with chicks at sunset.
26 Nov 2018	On arrival several dead chicks were observed, and part of a dead adult Fairy Tern was found at the edge of the sanctuary (part of head, bill and feathers). The remains appear to be at least a day old and was being eaten by ants. Residents from apartments opposite the sanctuary reported a white cat in the sanctuary at night. Two cat traps set by rangers and animal-control agent.
27 Nov 2018	Several chicks were found dead or missing, including the first chick to hatch (now ~8 days old). Cat tracks and cat scats were observed in and around the sanctuary. Wildlife cameras serviced and repositioned and sonic devices set-up around the nesting birds. Rangers continuing trapping. ~98 nests in the sanctuary; 42 nests on the beach.
28 Nov 2018	A letter was delivered to neighboring residents advising of the Fairy Tern deaths and requesting that pet cats be kept indoors. This prompted several community members to call and report observations of a prowling white cat in the area, which had also been seen on the rock wall adjoining the sanctuary. ~92 nests in sanctuary.
29 Nov 2018	Local residents found two dead Fairy Tern adults on a side-street. Both bodies were decapitated, and their breasts opened.
30 Nov 2018	Residents opposite the sanctuary reported a white cat in the sanctuary overnight-no chicks were observed alive. Wildlife cameras confirmed the presence of a white cat. A photo taken from a second-story apartment balcony show the cat in the sanctuary and it appears to be eating something. The sonic devices did not deter the cat from entering the sanctuary. A decapitated Fairy Tern carcass was retrieved by a resident from a nearby tree–it was being eaten by an Australian Raven, *Corvus coronoides*.
1 Dec 2018	Wildlife cameras took photos of a white cat in the sanctuary in the early hours of the morning-04:12 and 04:15 (Figure 2). Overnight monitoring of sanctuary and beach-nesting colony undertaken. At~19:00, a white cat was observed walking along a footpath towards the sanctuary. The cat was shooed away, and the cat took refuge in a construction site, but despite attempts to locate the cat, it could not be found. The cat returned to the sanctuary around midnight. It walked along the seawall towards the sanctuary but after being detected by observers it hid in the wall until it was, eventually, shooed away. Sometime after, the cat returned and entered the sanctuary. It was not detected until it was half way into fenced sanctuary area. The cat was in a crouch, stalked position slowing moving closer to the nesting birds, who were unaware of the cat’s presence. The cat was chased out of the sanctuary and followed (from a distance of~20 m) for~1 km in an attempt to understand where the cat was residing. The cat was eventually lost in coastal scrub.
2 Dec 2018	~92 nests in the sanctuary and 7 nests with chicks at sunset. No cats or other predators were seen during overnight monitoring.
3 Dec 2018	City of Mandurah sent out a second letter to neighboring residents requesting that cats be kept indoors. Overnight monitoring of sanctuary and beach-nesting colony. No cats observed
4 Dec 2018	Overnight monitoring of sanctuary and beach-nesting colony. No cats observed
5 Dec 2018	Overnight monitoring of sanctuary and beach-nesting colony. No cats observed
6 Dec 2018	Wildlife cameras took photos of a white cat in the sanctuary at 04:21 and 22:07.
7 Dec 2018	A registered, pet cat was trapped~1 km away from sanctuary and impounded.
8 Dec 2018	25 chicks were being fed by parents in the sanctuary. The adult birds were unsettled and spending little time on the ground tending to chicks. A Nankeen Kestrel, *Falco cenchroides,* depredated two chicks between 16:00 and 18:00. The terns appeared to have lost social cohesion. Resident found a decapitated adult tern carcass in the bush near the sanctuary.
11 Dec 2018	Residents reported seeing white cat in and around the sanctuary, which was subsequently chased away in the middle of the night. Multiple chicks killed, missing or injured and the carcasses of two decapitated adults were found in a nearby street. The colony was almost deserted by afternoon apart from a few adults coming to feed 7 chicks but not spending any time on the ground. A Nankeen Kestrel was observed depredating two of the remaining chicks.
12 Dec 2018	The white cat was captured–the cat was desexed but neither collared nor microchipped as required under relevant legislation (Western Australian Cat Act 2011 [36]).
16 Dec 2018	A Nankeen Kestrel depredated the last two chicks from the sanctuary. City of Mandurah volunteers removed chick shelters from the site.

**Table 3:** Timeline showing the development of the beach-nesting colony at Mandurah, disturbance and predation events, and management activities undertaken during the 2018–2019 breeding season (30 October 2018 to 31 December 2019).

Date	Timeline of Events
28 Oct 2018	Annual sand-by passing program ceased.
30 Oct 2018	Three Fairy Tern nests observed on the beach; 1 nest subsequently destroyed (eggs eaten) by Silver Gulls, *Chroicocephalus novaehollandiae.*
2 Nov 2018	~34 nests on the beach; unleashed dog on beach close to the colony.
7 Nov 2018	Two nests moved, incrementally, up the beach due to continued erosion.
11 Nov 2018	Beach eroding-corner post of roped area fallen down; ~20 nests on the beach.
15 Nov 2018	Human foot tracks leading into the colony but stopped near closest nest.
17 Nov 2018	Several nests abandoned close to water due to the beach eroding. New nests being made on higher ground.
18 Nov 2018	Human foot tracks leading through the middle of the colony. Children running close to the edge of the roped area.
19 Nov 2018	Beach continuing to erode; roped area on water side of colony re-adjusted.
23 Nov 2018	~40 nests on the beach.
26 Nov 2018	5 unleashed small dogs playing at the edge of the colony; birds unsettled. ~40 nests on beach.
29 Nov 2018	Large dog foot-tracks observed in the colony. Fairy Tern observed discarding an egg-shell (with small embryo inside) at the water’s edge (Corker, C. 2018, Fairy Tern Conservation Network, pers. comm.).
5 Dec 2018	First two chicks on the beach hatched. Chick-shelters installed to provide protection against aerial predators.
13 Dec 2018	One chick found 100 m down the beach; contractors installed shade cloth fencing around the colony to prevent incursions by humans and dogs. Rangers close off area between the sanctuary and the beach-colony to protect nesting birds.
16 Dec 2018	Nankeen Kestrel, *Falco cenchroides,* depredating chicks from the beach; 21 nests including 10 chicks. Several tern pairs moved chicks up on to the rock wall. Some nests were noted as having been abandoned.
23 Dec 2018	Several nests sighted on sea wall between beach colony and sanctuary.
26 Dec 2018	Nankeen Kestrel observed depredating chick from the beach.
31 Dec 2018	Chick-shelters removed from nesting area as site had been abandoned.
